# Cardiovascular risk prediction and influencing predictors identification among Bangladeshi individuals using machine learning algorithms and association rule mining

**DOI:** 10.1371/journal.pone.0333913

**Published:** 2025-10-07

**Authors:** Md. Merajul Islam, Sujit Kumar, Md. A. Salam, Dulal Chandra Roy, Md. Rezaul Karim

**Affiliations:** 1 Department of Statistics, Jatiya Kabi Kazi Nazrul Islam University, Mymensingh, Bangladesh; 2 Department of Statistics, University of Rajshahi, Rajshahi, Bangladesh; McMaster University, CANADA

## Abstract

**Background:**

Cardiovascular disease (CVD) encompasses a group of disorders that affect the heart and blood vessels, making it one of the leading causes of death globally, including in Bangladesh. Applying predictive modeling for the early identification and detection of CVD holds significant promise for saving lives by enhancing prediction precision through machine learning algorithms. Therefore, this study aimed to predict high-risk individuals for CVD using machine learning algorithms and identify its influencing predictors by association mining rules among individuals in Bangladesh.

**Materials and methods:**

This study utilized the most recent Bangladesh Demographic and Health Survey (BDHS) 2022 data, which encompassed 2,221 respondents. A Boruta-based feature selection method is employed to determine the important features associated with the high risk of CVD. Different machine learning algorithms, including logistic regression, Naïve Bayes, artificial neural network, random forest, and extreme gradient boosting (XGB), are adopted to predict the high-risk individuals for CVD in the training dataset. The predictive performance of the models is evaluated using accuracy, precision, recall, F1-score, and area under the curve (AUC) in the testing set. Additionally, the most significant rules are analyzed using the association mining technique to identify the influencing predictors of high risk of CVD.

**Results:**

The Boruta method indicated that age, residence, marital status, wealth, having an air conditioner (AC), and body mass index (BMI) are important predictors of high risk of CVD. The XGB-based predictive model achieves impressive performance compared to other models, with an accuracy of 68.22%, precision of 69.70%, F1-score of 79.54%, and AUC of 0.721. The association rules identified that being aged 65 or older, living in an urban area, having the richest wealth status, having AC, and being widowed are the influencing predictors of high risk of CVD.

**Conclusions:**

This study emphasizes the potential of XGB in predicting high-risk individuals for CVD and enhances the investigation of key factors contributing to CVD risk in this population, thereby facilitating the development of targeted prevention strategies that can effectively mitigate the high CVD risk.

## 1. Introduction

Cardiovascular diseases (CVD) represent a major global health concern, being one of the leading causes of morbidity and mortality worldwide [1]. These encompass a variety of conditions affecting the heart and blood vessels, such as heart failure, stroke, and coronary artery disease [2–4]. This category of diseases is particularly prevalent among middle-aged and older individuals, contributing to one-third of all deaths. Unfortunately, low-income and middle-income nations, such as Bangladesh, bear a disproportionately high incidence of deaths attributed to CVD [5]. The rise in deaths from CVD in Bangladesh over the past few decades is very worrying. In 1986, the death rate from CVD was 11 per 10,000 people, but by 2006, it had jumped to 411 per 10,000 people [6]. In 2018, around 0.256 million people in Bangladesh died from CVD [7]. The burden of CVD is not just a public health challenge but also an economic one [8]. It imposes significant financial strain on patients and their families due to the high costs of long-term treatment and care. Additionally, healthcare systems face immense pressure as they struggle to manage the rising number of CVD cases. This includes costs related to hospital admissions, medication, surgeries, and ongoing care, which further stretch already limited healthcare resources, particularly in low- and middle-income countries like Bangladesh. The economic impact of CVD makes it essential to prioritize investment in prevention and early detection to reduce the health and financial burden of these conditions. Early detection and prediction are key to reducing morbidity and mortality, particularly in high-risk groups such as patients with type-2 diabetes (T2D), who are more prone to developing CVD. Predicting high-risk individuals for CVD can transform healthcare by allowing providers to identify the individuals at risk before the disease fully develops. This enables timely interventions, such as lifestyle modifications, medication, or monitoring, which can significantly lower the chances of disease progression and lead to better patient outcomes. By focusing on prevention and early treatment, healthcare systems can not only improve the quality of life for patients but also reduce the long-term costs associated with treating advanced CVD. However, to effectively address the rising burden of CVD, advanced techniques must concentrate on identifying the key contributing factors. Furthermore, developing a predictive model that can predict the disease’s status early is crucial. Utilizing machine learning (ML) algorithms presents a highly promising approach for enhancing the precision and accuracy of CVD prediction. These algorithms are particularly well-suited for analyzing large and complex datasets, as they can effectively model non-linear relationships among variables. Moreover, ML techniques can detect subtle patterns and interactions that conventional statistical methods often overlook. Moreover, ML models performed well for identifying hidden patterns and interactions that traditional statistical methods often overlook.

In recent years, numerous studies have demonstrated the application of various ML techniques for diagnosing and predicting high-risk individuals for CVD [9–18]. While these approaches have shown considerable potential, their effectiveness has not been consistent across different populations and datasets. Importantly, despite the global rise in the adoption of ML in healthcare, there remains a notable research gap in Bangladesh. To the best of our knowledge, no existing study has comprehensively addressed the early detection of CVD among individuals using ML algorithms applied to the most recent nationally representative dataset. Additionally, previous research has largely overlooked the use of association rule mining—a powerful data mining technique capable of uncovering hidden relationships among variables—to explore the underlying interactions between key risk factors of CVD. This analytical gap limits our understanding of the combinatorial effects of predictors that may jointly influence CVD risk. Therefore, the present study aims to address these gaps through a twofold objective: (1) to develop the most suitable predictive model for predicting high-risk individuals for CVD, using machine learning algorithms; and (2) to identify influential predictors of CVD through association mining techniques. By integrating predictive modeling with association rule mining, this study not only enhances individual-level risk prediction but also contributes to identifying actionable predictors. This combined approach can inform more effective risk stratification and targeted public health interventions for reducing the burden of CVD in Bangladesh.

The organization of the remaining part of this work is structured as follows: Section 2 introduces the materials and methods utilized. The data analysis results are presented in Section 3, and a detailed discussion is provided in Section 4. Finally, the conclusions are given in Section 5.

## 2. Materials and methods

### 2.1. Data source

**Fig 1:**
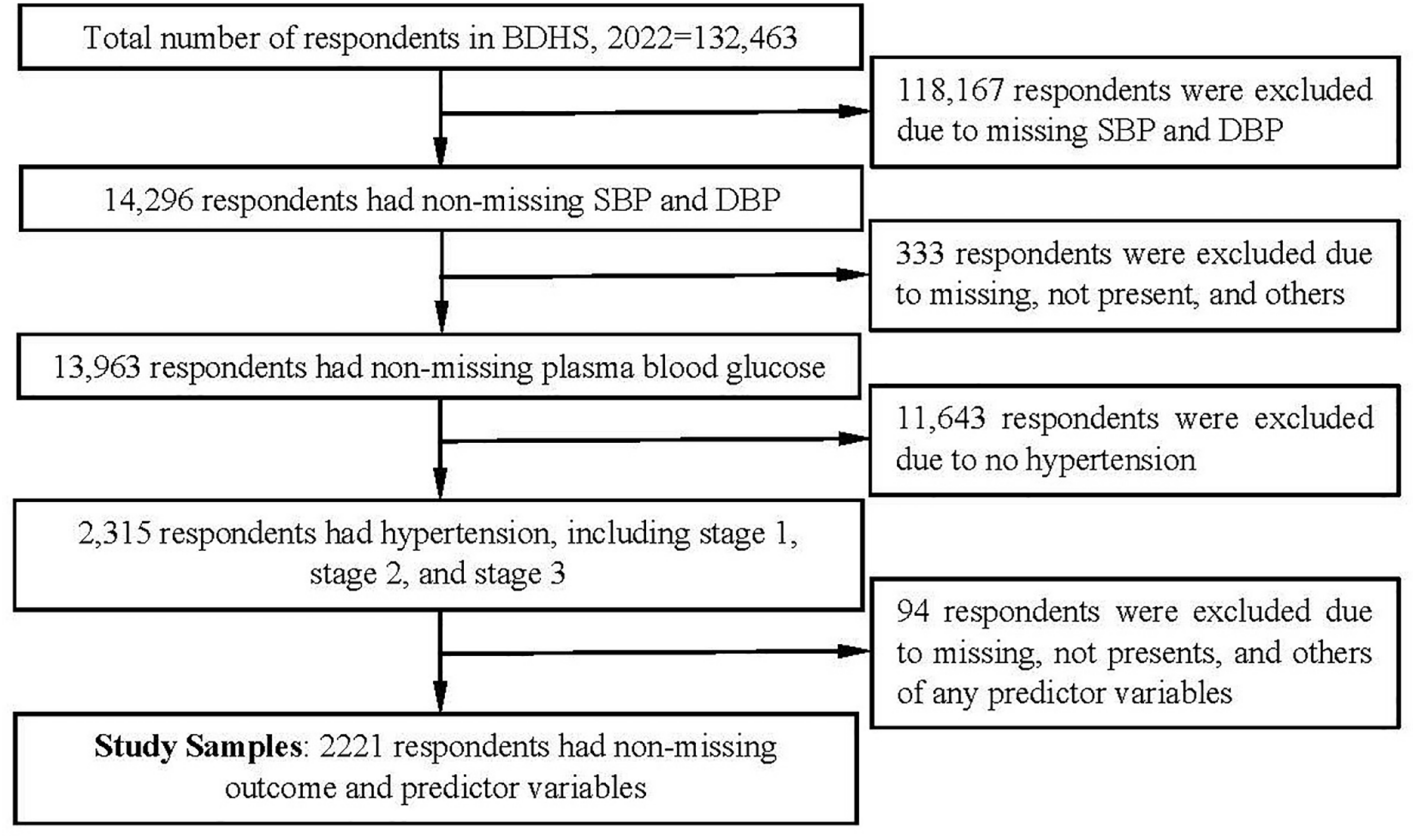
Sample selection flowchart.

The dataset for this study was derived from the 2022 Bangladesh Demographic and Health Survey (BDHS). The survey employed a two-stage stratified cluster sampling design for data collection [19]. In the first stage, enumeration areas (EAs) were selected using probability proportional to size sampling, consisting of 237 urban and 438 rural areas. In the second stage, a fixed number of households (typically around 30 per EA) are systematically selected from the designated EAs. Data are collected through face-to-face interviews using a structured questionnaire on various topics, including demographic characteristics, health indicators, nutrition, family planning, maternal and child health, and non-communicable diseases. The BDHS 2022 collected data from 30,330 households, with interviews completed in 30,018 of them, encompassing 132,463 individuals. Among these individuals, 118,167 were excluded due to missing data on systolic and diastolic blood pressure (SBP and DBP), resulting in a sample of 14,296 respondents. Subsequently, 333 individuals were excluded due to missing, not present, or other issues related to plasma blood glucose, resulting in 13,963 respondents with complete blood glucose data. From among them, 11,643 individuals without hypertension were excluded, yielding 2,315 respondents identified as having hypertension, including those with stage 1, stage 2, and stage 3. Finally, 94 respondents were eliminated due to missing, not present, or other issues related to any predictor variables, resulting in a study sample of 2,221 respondents. The sample selection procedure is presented in Fig 1.

### 2.2. Variable description

#### 2.2.1. Outcome variable.

The outcome variable of interest in this study is the high risk of CVD, which was constructed by combining information on individuals with hypertension and diabetes, using the WHO/ISH risk prediction guidelines [20,21]. Initially, the presence of hypertension was measured as if a respondent had a systolic blood pressure (SBP) ≥140 mmHg and/or a diastolic blood pressure (DBP) ≥90 mmHg [22,23]. This categorization was further classified into three different stages: Stage 1 (SBP 140 mmHg −159 mmHg or DBP 90 mmHg −99 mmHg), Stage 2 (SBP 160 mmHg −179 mmHg or DBP 100 mmHg −109 mmHg), and Stage 3 (SBP > 180 mmHg or DBP > 110 mmHg) [24,25]. We also determined that individuals with fasting plasma glucose levels of 7.0 mmol/L or higher were considered diabetic, and those with levels below this threshold were considered non-diabetic. We further categorized hypertensive patients into three risk groups (Das, et al., 2024) —low, medium, and high—based on the presence or absence of diabetes as follows.

- Type-2 diabetes absent:
  - Stage 1 hypertension (SBP 140–159 or DBP 90–99): Low risk
  - Stage 2 hypertension (SBP 160–179 or DBP 100–109): Medium risk
  - Stage 3 hypertension (SBP >180 or DBP >110): High risk
- Type-2 diabetes present:
  - Stage 1 hypertension (SBP 140–159 or DBP 90–99): Medium risk
  - Stage 2 hypertension (SBP 160–179 or DBP 100–109): High risk
  - Stage 3 hypertension (SBP >180 or DBP >110): High risk

This high-risk group includes those with diabetes and/or those falling into the higher blood pressure categories (Stage 2 or Stage 3). Individuals who meet either or both of these conditions are classified as high-risk for CVD as follows


$$\text{High risk of CVD}=\left\{ \begin{array}{ccc} \text{1}\;(\text{Yes}),\text{ if T2D present and}/\text{or fall into the Stage }\text{2}\text{ or }\text{3}\; & \; & \;\\ \text{0}\;(\text{No}),\text{Otherwise } \end{array}\right.\;$$ (1)

#### 2.2.2. Explanatory variables.

This study included various explanatory variables as predictors for a high risk of CVD, based on earlier studies and data availability in the BDHS 2022 database [26–32]. The variables are age, sex, division, residence, marital status, education, wealth, watching television, having a mobile, having a computer, having AC, coffee, smoking, and body mass index (BMI). BMI is categorized into four groups: underweight (BMI < 18.5 kg/m²), normal weight (18.5–24.9 kg/m²), overweight (25.0–29.9 kg/m²), and obese (BMI ≥ 30 kg/m²) [33].

**Ethical approval:** As the data is available to the public on their website, it is not required to obtain ethical review and approval for this research involving human participants in compliance with local laws and institutional regulations.

### 2.3. Statistical analysis

The characteristics of the study participants are reported as frequencies in percent (%). In the context of bivariate analysis, the Pearson $\chi^{\text{2}}$ test is employed to assess the relationship between the outcome and explanatory variables. The test was two-tailed, with a p-value<0.05 deemed as statistically significant. The dataset was then split into two sets, namely training and testing, using a random sampling with an 8:2 ratio [34]. The training set consisted of 1,778 respondents, while the testing set comprised 443 respondents. Statistical analysis was carried out using SPSS software (Version 27). The membership class label of the data was imbalanced (high-risk for CVD: 35.0% and non-high-risk for CVD: 65.0%). To address this problem, we applied the adaptive synthetic (ADASYN) oversampling technique, an improved version of the synthetic minority oversampling technique (SMOTE) that generates new minority class samples based on a weighted distribution [35].

#### 2.3.1. Feature selection.

Feature selection—also referred to as variable/attribute selection in statistics and machine learning—plays a critical role in creating an efficient prediction model by selecting the important features. Additionally, it can lead to shorter computation times, better generalization, higher interpretability, and improved model performance. To identify the important predictors of high risk of CVD, we executed the well-established Boruta feature selection method. The Boruta method is a wrapper-based feature selection technique that employs a random forest classifier to assess the importance of each feature [36,37]. In Boruta, the algorithm iteratively removes irrelevant features by comparing the significance of real features against random shadow features. Features that consistently perform worse than the shadow features are deemed irrelevant and are removed.

#### 2.3.2. Machine learning algorithms.

This study applied five popular machine learning (ML) algorithms to predict individuals at high risk for CVD. Below is a brief description of them:

#### 2.3.3. Logistic regression.

Logistic regression (LR) is a machine learning technique that predicts the probability of a binary outcome (i.e., yes/no) based on the input predictors [38]. It estimates the probability that a given input vector X is associated with a specific class (i.e., class 1) through the use of the logistic function. The logistic function is as follows


$$P\left(y=\text{1}|X\right)=\frac{\text{1}}{\text{1}+e^{-({\beta_{\text{0}}+\beta }_{\text{1}}x_{\text{1}}+\beta_{\text{2}}x_{\text{2}}+\ldots +\beta_{p}x_{p})}}$$ (2)

where, P(y=1|X) is the probability that the outcome y is 1 given the input features X, $\beta_{\text{0}}$ is intercept, and $\beta_{\text{1}}$, $\beta_{\text{2}}$, …, $\beta_{p}$ are the coefficients of the input predictors $x_{\text{1}},\;x_{\text{2}},\ldots ,\;x_{p}$, respectively. If P(y=1|X)≥0.5, the predicted class is 1; otherwise, it is 0.

#### 2.3.4. Naïve Bayes.

Naïve Bayes (NB) is a probabilistic classification algorithm that utilizes Bayes’ theorem, assuming that features are conditionally independent given the class label. NB often performs well despite this strong assumption of feature independence in many real-world applications, such as medical diagnostics like diabetes prediction [39]. The formula for NB classification is given as:


$$P\left(c|x_{\text{1}},\;x_{\text{2}},\ldots ,\;x_{p}\right)=\frac{P(c)\prod_{i=\text{1}}^{n}P(x_{i}|c)}{\prod_{i=\text{1}}^{n}P(x_{i})}$$ (3)

where, c is the outcome variable; $x_{\text{1}},\;x_{\text{2}},\ldots ,\;x_{p}$ are the input predictors; $P\left(c|x_{\text{1}},\;x_{\text{2}},\ldots ,\;x_{p}\right)$ is the posterior probability of class c given the predictors $x_{\text{1}},\;x_{\text{2}},\ldots ,\;x_{p}$; P(c) is the prior probability of class c; $P(x_{i}|c)$ is the conditional probability of predictors $x_{i}$ given class c; $P(x_{i})$ is the marginal probability of predictors $x_{i}$. To classify a new instance, NB picks the class c that maximizes the posterior probability:


$$c=\text{arg}\;\underset{c}{\text{max}}\;P(c)\;\prod_{i=\text{1}}^{n}P(x_{i}|c)$$ (4)

#### 2.3.5. Artificial neural network.

Artificial neural network (ANN) is a widely used machine learning algorithm capable of performing various tasks, including classification. It consists of interconnected nodes, called neurons, which are arranged into three layers: an input layer, one or more hidden layers, and an output layer. During training, the neural network adjusts the weights and biases of each neuron to minimize the difference between predicted and actual outputs. This is achieved using an optimization algorithm, such as gradient descent, which iteratively updates the weights and biases with the sigmoid activation function [40]. The sigmoid function, also known as the logistic function, is a widely used activation function in ANNs for binary classification. The sigmoid function formula is as follows


$$\sigma (z)=\frac{\text{1}}{\text{1}+e^{-z}}$$ (5)

where, z is the input. The procedure continues until the minimum error is reached or the iteration values remain unchanged.

#### 2.3.6. Random forest.

Random forest (RF) is a widely utilized machine learning algorithm for classification and regression tasks. It is an ensemble learning technique combining multiple decision trees by creating multiple subsets of the original training data through bootstrapping [41]. Let $D=\{\left(x_{\text{1}},y_{\text{1}}\right),\;\left(x_{\text{2}},y_{\text{2}}\right),\ldots ,(x_{N,\;}y_{N})$} be the training dataset, then the bootstrap sample $D_{i}$ for the $i^{th}$ tree can be represented as


$${\;D}_{i}=\{\left(x_{\text{1}i},y_{\text{1}}\right),\;\left(x_{\text{2}i},y_{\text{2}}\right),\ldots ,\left(x_{Ni,\;}y_{N}\right)\}$$ (6)

Each decision tree $T_{i}$ in the forest makes the output a predicted class label ${\hat{y}}_{i}$. The final prediction $\hat{y}$ for an input x is made by the majority voting:


$$\hat{y}=mode(\;{\hat{y}}_{\text{1}},\;{\hat{y}}_{\text{2}},\ldots ,\;{\hat{y}}_{B})$$ (7)

where, B is the total number of trees in the forest.

#### 2.3.7. Extreme gradient boosting.

Extreme gradient boosting (XGB) is a popular variant of gradient boosting that is used extensively for prediction due to its efficiency and predictive power. It works by iteratively improving the model’s predictions using decision trees as weak classifiers, each aiming to correct the errors of the preceding trees [42]. XGB typically uses logistic loss (log loss) to measure the difference between predicted probabilities and actual binary labels


$$L\left(y_{i},{\hat{y}}_{i}\right)=-\left[y_{i}\text{log}\left({\hat{y}}_{i}\right)+\left(\text{1}-y_{i}\right)\text{log}(\text{1}-{\hat{y}}_{i})\right]$$ (8)

where, $y_{i}$ is the true class label of the ith sample and $p_{i}$ is the predicted probability that the ith sample belongs to the positive class. In XGB, the raw prediction score (also called the logit) is computed as the sum of the outputs from all trees. For an input x, if there are T trees in the model, the raw prediction score $\hat{y}$ is given by:


$$\hat{y}=\sum_{i=\text{1}}^{T}T_{i}(x)$$ (9)

where, $T_{i}(x)$ is the output of the $i^{th}$ tree for the input x. The logistic function converts the raw prediction score into a probability. The logistic function is


$$P\left(y=\text{1}|x\right)=\frac{\text{1}}{\text{1}+e^{-(\sum_{i=\text{1}}^{T}T_{i}\left(x)\right)}}$$ (10)

where, P(y=1|x) is the probability that the outcome y is 1, given the input predictors X. If P(y=1|x)≥0.5, the predicted class is 1; otherwise, it is 0.

#### 2.3.8. Hyper-parameter tuning.

Hyperparameters are used to control the learning process of machine learning models. Unlike model parameters, which are learned during training, hyperparameters are set before the training process and can significantly influence the model’s performance. It is not easy to know what values to use for the hyper-parameters of a given algorithm on a given dataset, so random or grid searches for hyper-parameter values are commonly used techniques. In this study, hyperparameter tuning was performed using grid search methods, and the model was trained on the training data through 10-fold cross-validation.

#### 2.3.9. Model evaluation.

After training the model, model evaluation determines how the model performs by measuring the performance on previously reserved test datasets. The confusion matrix is a simple table that compares actual and predicted categories for the outcome variable (Table 1). True Positives (TP) are the positive cases correctly predicted as positive. True Negatives (TN) are the negative cases accurately predicted as negative. False Negatives (FN) are the positive cases incorrectly predicted as negative, while False Positives (FP) are the negative cases incorrectly predicted as positive.

**Table 1:** Confusion matrix.

		Prediction	
		Positive	Negative
Actual	Positive	True positive	False negative
	Negative	False positive	True negative

**Accuracy:** Accuracy is the ratio of the total number of correctly classified positive and negative instances to the overall number of instances. This can be expressed mathematically as follows:


$$Accuracy=\frac{TP+TN}{TP+FN+FP+TN}$$ (11)

**Precision**: Precision is the ratio of true positive predictions, accurately identified positive instances, to the total number of positive predictions generated by the model. This can be expressed mathematically as follows:


$$Precision=\frac{TP}{TP+FP}$$ (12)

**Specificity:** Specificity is the proportion of correctly predicted negative instances to the total actual negative instances. This can be expressed mathematically as follows:


$$Specificity=\frac{TN}{TN+FP}$$ (13)

**Recall**: Recall is the ratio of correctly predicted positive instances to the total actual positive instances. It is computed as follows:


$$Recall=\frac{TP}{TP+FN}$$ (14)

$F_{1}$**-score**: F1-score is the harmonic mean of precision and recall. This can be expressed mathematically as follows:


$$F_{\text{1}}-score=\frac{\text{2}(Precision*Recall)}{Precision+Recall}$$ (15)

#### 2.3.10. ROC curve.

The ROC curve is a graphical representation used to show the diagnostic effectiveness of a binary classifier as its discrimination threshold is varied [43]. It plots sensitivity against 1-specificity across different thresholds. The AUC, or area under the ROC curve, quantifies the performance of the model. A higher AUC reflects a more effective model, indicating better discrimination between positive and negative cases. We have used the caret package (version 7.0−1) in R to implement each classifier for model training, tuning, and performance evaluation.

#### 2.3.11. Association rules.

Association rule mining is a data mining technique that discovers interesting relationships or patterns in a dataset. It focuses on identifying associations or dependencies between items or events that occur frequently together in a given dataset and was first introduced by Agrawal et al. [44]. Association rules are typically represented as “if-then” statements or implications. The “if” part is called the antecedent or left-hand side (LHS) of the rule, while the “then” part is called the consequent or right-hand side (RHS) of the rule. The strength or quality of an association rule is usually measured in terms of support, confidence, and lift. Support indicates the proportion of transactions in the dataset that contain both the antecedent and the consequent of a rule. Higher support values imply that the rule occurs more frequently. Confidence measures the conditional probability of the consequent given the antecedent. A confidence of 1 indicates that the consequent always occurs when the antecedent is present. Lift assesses the significance of the association between the antecedent and the consequent. This study incorporated an additional approach that supports the classification of machine learning algorithms using the Apriori algorithm [45] to uncover the associations between the identified predictors and the outcome variable. The minimum support degree of 0.0011 and minimum confidence level of 90% were adopted to identify all potential association rules. A rule is considered reliable if its confidence level exceeds 80% [46]. This study focused on features implied by the outcome variable (Antecedent => Consequent), which is a method for classifying all predictors contributing to high-risk of CVD. These are commonly known as classification association rules [47]. This method determined the predictors that each category contributed to the outcome variable.

For a specific rule, the equations for support, confidence, and lift can be defined as follows: In this case, the feature sets represented by X and Y are mutually exclusive.


Rule X⇒Y (16)

$$Support=\frac{frequency(\text{X},\text{Y})}{N}$$ (17)

$$Confidence\;=\frac{\text{Support}(\text{X},\text{Y})}{\text{Support}(\text{X})}$$ (18)

$$\text{Lift }=\frac{frequency(\text{X},\text{Y})}{frequency(\text{X})frequency(\text{Y})}$$ (19)

**Fig 2:**
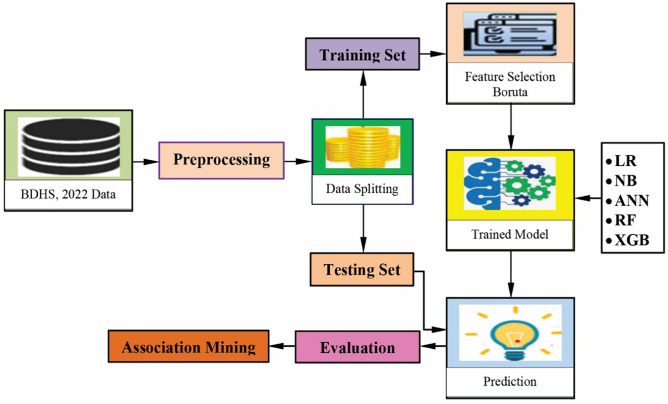
The workflow of this study.

We used the arules (Version: 1.7–7) package in R to perform association rule mining. The overall workflow of the study is illustrated in Fig 2.

## 3. Data analysis results

### 3.1. Basic characteristics

The basic characteristics of the respondents are presented in Table 2. The overall prevalence of high-risk of CVD is 35.0%. As shown in Table 2, the rural areas had the most significant representation, with 62.8% of respondents living there. The majority of respondents were female, comprising 62.9% of the sample. Of the respondents, 77.4% were married, and around 36.0% had no education. The participants aged ≥ 65 years showed the highest prevalence (41.5%) of high risk of CVD, while the age group ranging from 18 to 34 years showed the lowest prevalence (19.5%). The richest respondents had the highest prevalence (42.5%) of high-risk for CVD, while respondents with middle-class and lower-class socioeconomic status had lower prevalence. Respondents who watched television exhibited a larger proportion (38.0%) of high-risk for CVD. Among the categories related to body weight, overweight respondents exhibited a higher prevalence of high-risk for CVD at 36.8%, while the category of underweight respondents showed a lower prevalence (24.5%). Age, residence, marital status, wealth, watching television, having a mobile, having a computer, having AC, and BMI are associated with a high risk of CVD (p-value < 0.05).

**Table 2:** Basic characteristics of the respondents.

Predictors	Overall, n (%)	High-risk of CVD status		p-value
		No, n(%)	Yes, n(%)	
**Total**	2221(100)	1443(65.0)	778(35.0)	
**Age**				
18-34	354(15.9)	285(80.5)	69(19.5)	<0.001
35-44	450(30.3)	306(68.0)	144(32.0)	
45-64	947(42.6)	577(60.9)	370(39.1)	
≥ 65	470(21.2)	275(58.5)	195(41.5)	
**Sex**				
Male	830(37.4)	549(66.1)	281(33.9)	0.370
Female	1391(62.6)	894(64.3)	497(35.7)	
**Division**				
Barishal	224(10.1)	154(68.8)	70(31.3)	0.151
Chattogram	293(13.2)	187(63.8)	106(36.2)	
Dhaka	273(12.3)	165(60.4)	108(39.6)	
Khulna	302(13.6)	201(66.6)	101(33.4)	
Mymensingh	220(9.9)	143(65.0)	77(35.0)	
Rajshahi	360(16.2)	230(63.9)	130(36.1)	
Rangpur	314(14.1)	221(70.4)	93(29.6)	
Sylhet	235(10.6)	142(60.4)	93(39.6)	
**Residence**				
Urban	826(37.2)	490(59.3)	336(40.7)	<0.001
Rural	1395(62.8)	953(68.3)	442(31.7)	
**Marital status**				
Never married	76(3.4)	64(84.2)	12(15.8)	<0.001
Married	1720(77.4)	1136(66.0)	584(34.0)	
Widowed	387(17.4)	216(55.8)	171(44.2)	
Divorced	38(1.7)	27(71.1)	11(28.9)	
**Education**				
No education	799(36.0)	510(63.8)	289(36.2)	0.591
Primary	556(25.0)	361(64.9)	195(35.1)	
Secondary	585(26.3)	393(67.2)	192(32.8)	
Higher	281(12.7)	179(63.7)	102(36.3)	
**Wealth**				
Poorest	319(14.4)	216(67.7)	103(32.3)	<0.001
Poorer	408(18.4)	292(71.6)	116(28.4)	
Middle	414(18.6)	282(68.1)	132(31.9)	
Richer	475(21.4)	305(64.2)	170(35.8)	
Richest	605(27.2)	348(57.5)	257(42.5)	
**Household members**				
≤ 4	1123(50.6)	728(64.8)	395(35.2)	0.885
> 4	1098(49.4)	715(65.1)	383(34.9)	
**Watching television**				
No	987(44.4)	678(68.7)	309(31.3)	0.001
Yes	1234(55.6)	765(62.0)	469(38.0)	
**Having mobile**				
No	761(34.3)	492(64.7)	269(35.3)	0.031
Yes, smart	387(17.4)	268(69.3)	119(30.7)	
Yes, basic	974(43.9)	630(64.7)	344(35.5)	
Yes, both smart and basic	99(4.5)	53(53.5)	46(46.5)	
**Having computer**				
No	1997(89.9)	1313(65.7)	684(34.3)	0.022
Yes	224(10.1)	130(58.0)	94(42.0)	
**Having AC**				
No	2189(98.6)	1431(65.4)	758(34.6)	0.002
Yes	32(1.4)	12(37.5)	20(62.5)	
**Caffeinated drink**				
No	2056(92.6)	1338(65.1)	718(34.9)	0.709
Yes	165(7.4)	105(63.6)	60(36.4)	
**Smoking**				
No	2005(90.3)	1307(65.2)	698(34.8)	0.515
Yes	216(9.7)	136(63.0)	80(37.0)	
**BMI**				
Underweight	208(9.4)	157(75.5)	51(24.5)	<0.001
Normal	1064(47.9)	701(65.9)	363(34.1)	
Overweight	733(33.0)	463(63.2)	270(36.8)	
Obese	216(9.7)	1443(65.0)	778(35.0)	

### 3.2. Important predictors of high-risk for CVD

**Fig 3:**
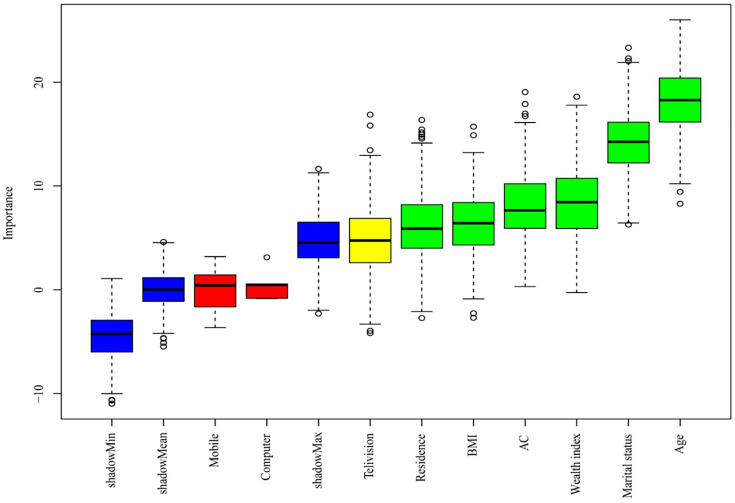
Important predictors of high-risk for CVD.

Fig 3 illustrates the outcomes of the Boruta feature selection method. The Boruta method identified age, residence, marital status, wealth, having AC, and BMI as significant predictors of high-risk individuals for CVD. The identified predictors were incorporated as risk factors in the development of ML models for predicting high-risk individuals for CVD.

### 3.3. Performance comparison of the models

The comparative performance of the ML-based models is presented in Table 3. The results indicated that the XGB model achieved an impressive predictive accuracy of 68.22% (95% confidence interval (CI): 66.03–70.30), precision of 69.70%, specificity of 55.39%, and F1-score of 79.54%. However, the RF-based model showed the highest recall of 96.74%.

**Table 3:** Performance comparison of ML-based models.

Models	Accuracy (%) (95% CI)	Precision (%)	Specificity (%)	Recall (%)	*F*_*1*_-score (%)
LR	65.69 (63.43-67.90)	66.87	53.99	93.51	77.98
NB	65.35 (63.09-67.57)	65.35	53.79	92.81	76.70
ANN	65.64 (63.33-67.80)	66.82	53.83	93.77	78.03
RF	66.26 (63.30-67.64)	65.49	54.62	96.74	78.11
XGB	68.22 (66.03-70.30)	69.70	55.39	92.61	79.54

**Fig 4:**
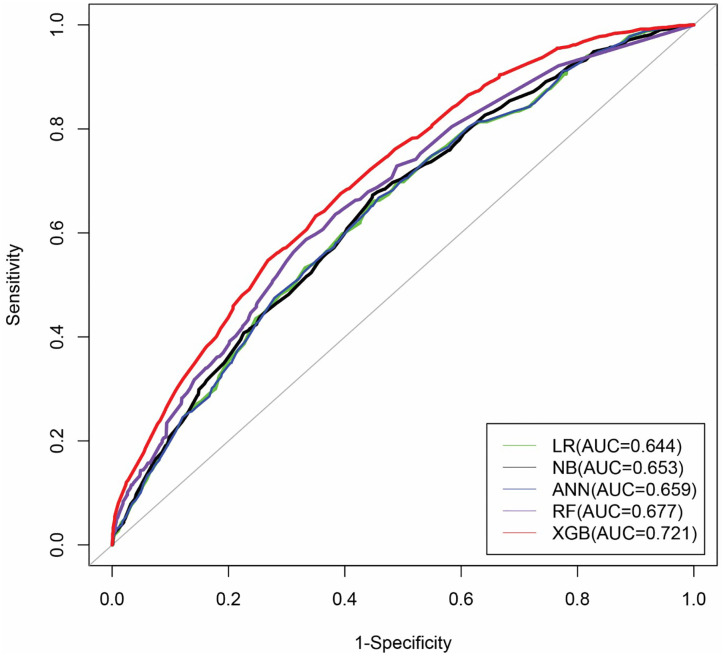
ROC curves of the models based on the BDHS, 2022 dataset.

The corresponding ROC curves for the machine learning models are presented in Fig 4. The ROC analysis also reveals that the XGB model attained the highest AUC value of 0.721, surpassing all other models. Thus, the findings demonstrated that the XGB-based model outperformed other models in predicting high-risk individuals for CVD.

### 3.4. Validation of the proposed model

To validate the performance of the proposed model, we utilized the well-established Framingham Heart Study dataset, which contains 4,240 patient records with 15 explanatory variables [48]. The dataset contained missing values; after excluding these, the final dataset comprised 3,658 individuals, including 557 CVD cases (15.2%) and 3,101 non-CVD cases (84.8%). We applied the same analytical protocol as used with the BDHS, 2022 data. The model’s performance on the Framingham dataset is presented in Table 4. Notably, the XGB model achieved the highest performance, with an accuracy of 86.25%, an F1-score of 89.03%, and an AUC of 0.801.

**Table 4:** Validation of the proposed model using the Framingham Heart dataset.

Models	Accuracy (%) (95% CI)	Precision (%)	Specificity (%)	Recall (%)	*F*_*1*_-score (%)
LR	85.51(84.21-86.83)	72.52	99.41	85.84	78.71
NB	82.00(80.62-83.41)	72.15	92.53	87.13	78.97
ANN	85.62(84.35-86.90)	70.53	99.34	85.91	77.46
RF	85.71(84.40-86.91)	72.90	99.82	85.91	78.85
XGB	86.25 (84.90-87.51)	92.21	99.46	86.15	89.03

**Fig 5:**
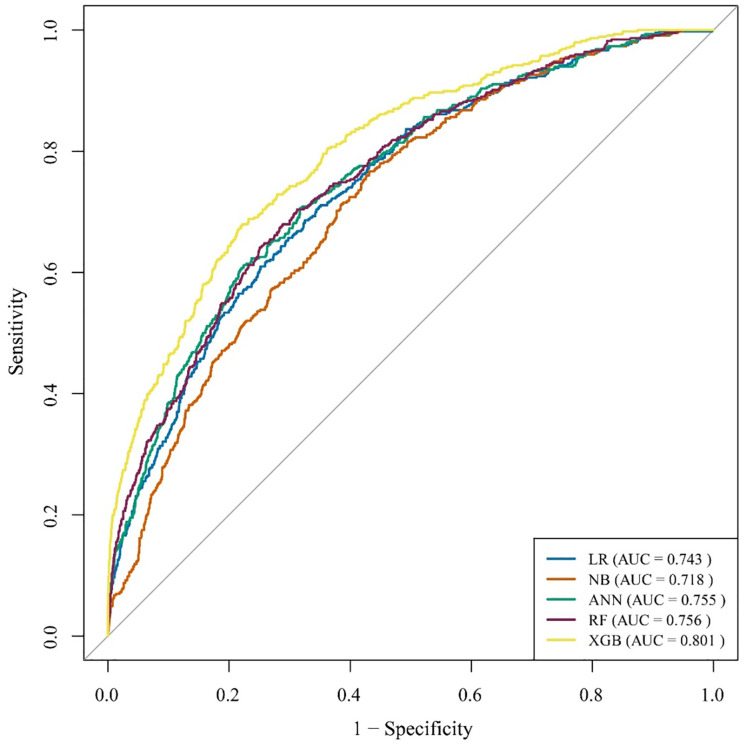
ROC curve of the models on the Framingham dataset.

The corresponding ROC curve is depicted in Fig 5. The XGB model showed the highest area in the ROC curve compared to other models, indicating superior discriminatory ability. Therefore, we propose that the predictive model demonstrates the highest performance across both the BDHS 2022 and Framingham datasets, supporting its potential generalizability and applicability in broader settings.

### 3.5. Association rules of high-risk for CVD

This study employed association rule mining using the Apriori algorithm to uncover meaningful patterns and relationships among the most relevant predictors, which were initially selected using the Boruta feature selection method. This approach was chosen to identify combinations of factors that frequently co-occur with high-risk individuals for CVD, offering deeper insights beyond individual predictor effects. As a result, 13 association rules with a confidence level of over 90% and the highest lift values were identified. Of all the rules, the top five most important rules were selected for predicting high-risk individuals for CVD, as illustrated in Table 5.

**Table 5:** Important association rules to predict high-risk individuals for CVD.

SN	Rules	Support	Confidence	Lift
1	{Residence = Urban, Wealth index = Richest, Having AC = Yes} => {High risk of CVD = Yes}	0.015	0.963	2.01
2	{Residence = Urban, Wealth index = Richest, Having AC = Yes, Marital status = Widowed} => {High risk of CVD = Yes}	0.012	0.957	1.99
3	{Having AC = Yes, BMI = Overweight} => {High risk of CVD = Yes}	0.012	0.955	1.99
4	{Wealth index = Richest, Having AC = Yes, BMI = Overweight} => {High risk of CVD = Yes}	0.012	0.955	1.99
5	{Residence = Urban, Wealth index = richest, Age ≥ 65, BMI = Overweight} => {High risk of CVD = Yes}	0.011	0.944	1.98

**Rule 1**: states that if the studied participants were urban residents with the richest wealth and living with AC, then the possibility of developing a high-risk individuals for CVD is 96.3% confidence.

**Rule 2**: indicates that if the studied participants were urban residents, the richest wealth, living with having AC, and those who are widowed, then the possibility of developing a high-risk individuals for CVD is 95.7% confidence.

**Rule 3** states that if study participants have AC and an overweight BMI, the possibility of having high-risk individuals for CVD is 95.5% confidence.

**Rule 4** means that if the participants are the richest in wealth, have an AC, and have an overweight BMI, then the possibility of having high-risk individuals for CVD is 95.5% confidence.

**Rule 5** shows that if the studied participants residing in urban areas with the richest wealth, have an age ≥ 65 years, and are overweight, then the possibility of having high-risk individuals for CVD is 94.4% confidence.

The results of the association rule suggest that the factors – being aged 65 or older, urban living, higher socioeconomic status, having AC, and being widowed, are important predictors of high-risk individuals for CVD (lift value>1.5). The discovery of strong association rules enhances understanding of underlying relationships between variables, which can inform clinical practices and policy decisions.

## 4. Discussion

CVD, a condition with high heritability, is a leading cause of death globally. Despite the high global prevalence of CVD, the level of awareness regarding this condition remains significantly low [49]. However, this study tried to predict high-risk individuals for CVD and identify its co-occurring influencing predictors using machine learning and association rule mining. Initially, the Boruta feature selection technique was employed to determine predictors of high-risk individuals for CVD. The analysis revealed that age, place of residence, marital status, wealth, having an AC, and BMI are the important predictors of high-risk individuals for CVD. We subsequently applied several machine learning algorithms to predict high-risk individuals for CVD. Among them, the XGB models exhibited superior performance compared to the other models. Numerous studies have developed models to predict high-risk individuals for CVD across different countries, utilizing diverse datasets and employing various statistical and machine learning algorithms [50–55] (S1 Table). The findings of this study differ from those reported in earlier studies due to variations in sample size, predictor variables, geographical and demographic contexts, and other factors. Finally, the association rule mining is employed to analyze and identify combinations of predictors that frequently appear together and are associated with the outcome. This helps to understand better the interactions between multiple factors rather than just single predictors. The association rules identified the most significant predictors of high CVD risk as individuals aged 65 years or older, urban residents, widowed individuals, those from the wealthiest families, people with access to AC, and those who are overweight. The results of this study suggest that age, especially older age, is the most crucial predictor of CVD, consistent with earlier studies [56,57]. This relationship is attributed to its association with obesity, chronic inflammation, and oxidative stress, all of which may elevate the risk of heart-related conditions. The highest prevalence of CVD has been observed in urban regions, where rapid urbanization often leads to a more sedentary lifestyle [58]. In urban settings, individuals are more likely to engage in desk-bound jobs and rely on vehicles for transportation, which results in reduced physical activity. This shift towards a sedentary lifestyle is compounded by factors such as increased availability of fast food, higher levels of stress, and environmental pollutants, all of which contribute to elevated risks for CVD. Moreover, the lack of green spaces and recreational areas in many urban environments further limits opportunities for exercise, making it essential to address these lifestyle changes through public health initiatives and urban planning to mitigate the impact of CVD in these populations. This study found that marital status is an associated predictor of CVD, which is consistent with findings from previous studies [57]. Studies have shown that unmarried individuals, especially those who are widowed, often exhibit a higher risk of CVD [59,60]. This association may be attributed to factors such as increased emotional stress, social isolation, and lifestyle changes that can occur following significant relationship changes, ultimately impacting cardiovascular health. Another important predictor identified in our study is wealth, particularly among the richest families, which is positively associated with a high risk of CVD [61–63]. While the richest wealth status is often associated with better access to healthcare and healthier lifestyle choices, it can also lead to increased stress levels, unhealthy dietary habits, and sedentary behaviors that contribute to a high risk of CVD. Given their busy lifestyles, wealthier individuals may have access to more processed foods and engage in less physical activity. Furthermore, the psychological stress associated with maintaining a high social and economic status can adversely affect cardiovascular health. Understanding this correlation is crucial for developing targeted interventions to address high CVD risk across different socioeconomic groups.

We found that having AC is an important predictor of CVD, consistent with previous research indicating the role of environmental factors in cardiovascular health [64–66]. Access to AC can help regulate indoor temperatures, reduce humidity, and improve air quality, all contributing to a healthier living environment. Urban environments, where AC use is more common, are frequently characterized by more sedentary lifestyles due to limited opportunities for outdoor physical activity, increased reliance on mechanized transportation, and occupational structures that involve prolonged sitting. Sedentary behavior has been robustly linked to elevated risks of hypertension, obesity, and other cardiometabolic conditions, all of which are established precursors to CVD [67,68]. Moreover, prolonged use of AC may lead to increased time spent indoors, which could reduce exposure to natural ventilation and less physical activity. Moreover, urban dwellers who rely on AC may be exposed to higher levels of indoor air pollutants, particularly in settings where ventilation is poor or where AC systems are not regularly maintained. Additionally, AC access may indicate greater exposure to urban outdoor air pollution, including nitrogen dioxide (NO₂) and ozone (O₃), which have been consistently associated with increased cardiovascular morbidity and mortality [69,70]. These pollutants are known to promote endothelial dysfunction, oxidative stress, and systemic inflammation—pathways that play critical roles in the pathogenesis of CVD [71]. This study has also shown that BMI, particularly being overweight, is a significant predictor of high-risk individuals for CVD. Elevated BMI is associated with increased body fat, which can lead to various health issues, including hypertension, dyslipidemia, and insulin resistance – all of which are major risk factors for high CVD risk. Overweight individuals often experience increased strain on the cardiovascular system, contributing to higher rates of heart disease and related complications [72,73]. Addressing overweight and obesity through lifestyle modifications, such as improved diet and increased physical activity, is crucial for reducing high CVD risk and promoting overall cardiovascular health. The identified important features can guide the development of targeted interventions. Raising awareness about healthy lifestyles, especially for older adults, is essential in mitigating the high risk of CVD. Targeted initiatives in key regions can strengthen health systems and increase awareness, which helps reduce the incidence of high CVD risk in these communities.

### 4.1. Limitations and future works

This study has several limitations that should be considered. First, the BDHS data used in this study is cross-sectional, which prevents us from establishing causal relationships between the predictors and the outcome variable. Second, the data used were primarily based on self-reported responses, which may be subject to recall bias and social desirability bias. Participants might have underreported or overreported certain behaviors or health conditions, leading to potential misclassification. Third, the findings may not be generalizable to populations outside the survey setting or to individuals not included in the BDHS sample, such as those who are institutionalized or homeless. Fourth, some variables, such as having AC or watching television, were used as proxies for a sedentary lifestyle, which may not fully capture actual physical inactivity and could introduce measurement bias. To address these limitations, future studies should utilize longitudinal data to explore cause-and-effect relationships and changes over time in high-risk individuals for CVD. Incorporating reliable and standardized measures ensures a more accurate evaluation of a sedentary lifestyle. Additionally, incorporating more clinical variables—such as cholesterol levels, blood glucose, HbA1c, waist-hip ratio, alcohol use, and family history of CVD could improve the model’s accuracy and provide a better understanding of what influences the high-risk for CVD.

## 5. Conclusion

Several models were applied during experimentation to predict the high risk of CVD. Among them, the XGB model, which utilized predictors selected by the Boruta method, demonstrated superior performance compared to the other models. The selected predictors of CVD were employed in association rule mining to investigate their patterns and interrelations with high-risk individuals for CVD. Finally, the analysis identified that age ≥ 65 years, urban residence, richest wealth, having AC, and overweight BMI were the most associated predictors with higher confidence of CVD. This highlights their potential as reliable decision-support tools, enabling healthcare professionals, policymakers, and other stakeholders to make informed decisions for early intervention and personalized treatment strategies. Ultimately, this can improve cardiovascular health outcomes and help reduce the growing mortality rate and healthcare burden in Bangladesh.

## Supporting information

S1 Table Comparative model performance with existing risk models. (DOCX)
