# Supplementary material for: Cardiovascular risk prediction and influencing predictors identification among Bangladeshi individuals using machine learning algorithms and association rule mining
Source: PLoS One. 2025 Oct 7;20(10):e0333913. doi: 10.1371/journal.pone.0333913 (PMC12503297; doi:10.1371/journal.pone.0333913)
Supplement: S1 Table — (DOCX) [file pone.0333913.s001.docx]

**Table S1**. **Comparative model performance** with **existing risk models**

| **Author & Year** | **Dataset & Sample Size** | **Feature Selection** | **Machine Learning Algorithms** | **Performance Metrics** | **Best Model & Performance** | **Association Rule Mining** |
| --- | --- | --- | --- | --- | --- | --- |
| Sianga et al. (2025) | Hospital (3553) | Chi-square and Information gain | SVM, NB, LR, XGB, RF, DT | Accuracy, F1-score, AUC | XGB  Accuracy of 95.24 | No |
| Shah et al. (2025) | **Hospital (10,000)** | SHAP, t-SNE, PCA | Gradient Boosting, CatBoost, LightGBM, SVM, and Neural Network | Accuracy, F1-score, AUC | XGB  Accuracy of 82%, F1-score of 82%, AUC of 0.82 | No |
| Hossain et al. (2024) | Bangladesh (651) | Chi-square, SHAP (via feature importance plot) | LR, NB, DT, AdaBoost, RF, Bagging Tree, ensemble | Accuracy, F1-score, AUC | RF  Accuracy of 98.04%, F1-score of 97.7%, AUC of 0.989 | No |
| Saeedbakhsh et al. (2023) | Isfahan Cohort (11,495) | Not specified | SVM, ANN, RF | Accuracy | SVM  Accuracy of 89.7% | Yes |
| Theerthagiri et al. (2022) | **Electronic health record (**70000) | RFE | LR, RF, MLP, SVM, NB, GB | Accuracy, F1-score, AUC | GB  Accuracy of 0.85 | No |
| Dinesh et al. (2018) | UCI Heart Disease (Cleveland) – 303 | PCA | LR, RF, SVM, DT, KNN, NB, GB, XGB | Accuracy, F1-Score, AUC | XGB: Accuracy 85%, F1 84.5%, AUC of 0.87 | No |
| Current study | Bangladesh (2,221) | BDHS | LR, NB, ANN, RF, XGB | Accuracy, F1-score, AUC | XGB  Accuracy of 68.22%, AUC of 0.721 | Yes |

**References**

1. Sianga BE, Mbago MC, Msengwa AS. PREDICTING THE PREVALENCE OF CARDIOVASCULAR DISEASES USING MACHINE LEARNING ALGORITHMS. Intelligence-Based Medicine. 2025 Jan 20:100199.
2. Shah P, Shukla M, Dholakia NH, Gupta H. Predicting cardiovascular risk with hybrid ensemble learning and explainable AI. Scientific Reports. 2025 May 23;15(1):1-21.
3. Hossain S, Hasan MK, Faruk MO, Aktar N, Hossain R, Hossain K. Machine learning approach for predicting cardiovascular disease in Bangladesh: evidence from a cross-sectional study in 2023. BMC Cardiovascular Disorders. 2024 Apr 18;24(1):214.
4. Kavya Reddy Jinne, Srinath Reddy Kandula and Wisam Bukaita*. Cardiovascular Disease Prediction Using Machine Learning. Am J Biomed Sci & Res. 2025 - 27(2). AJBSR.MS.ID.003539. DOI:[10.34297/AJBSR.2025.27.003539](http://dx.doi.org/10.34297/AJBSR.2025.27.003539)
5. Theerthagiri P. Predictive analysis of cardiovascular disease using gradient boosting based learning and recursive feature elimination technique. Intelligent Systems with Applications. 2022 Nov 1;16:200121.
6. Dinesh KG, Arumugaraj K, Santhosh KD, Mareeswari V. Prediction of cardiovascular disease using machine learning algorithms. In 2018 international conference on current trends towards converging technologies (ICCTCT) 2018 Mar 1 (pp. 1-7). IEEE.
